# Molecular biology and design research: A new paradigm and transdisciplinary approach to communicating science

**DOI:** 10.1038/s44319-025-00434-4

**Published:** 2025-03-31

**Authors:** Carla Molins-Pitarch, Jonas Krebs, David Brena

**Affiliations:** 1 Image Processing and Multimedia Technology Center, Universitat Politècnica de Catalunya-Barcelona Tech, Terrassa, Spain; 2 Elisava, Barcelona School of Design and Engineering (UVic-UCC), 08002 Barcelona, Spain; 3 Centre for Genomic Regulation (CRG), The Barcelona Institute of Science and Technology, Dr. Aiguader 88, Barcelona, 08003 Spain; 4 Universitat Pompeu Fabra (UPF), Barcelona, Spain

**Keywords:** Careers, Chromatin, Transcription & Genomics, Methods & Resources

## Abstract

The transdisciplinary ChromDesign project explored and demonstrated the power of Design research for developing new tools for visualizing and communicating complex concepts in molecular biology to diverse audiences.

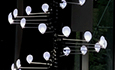

Research projects are built on diligent planning: defining clear aims, formulating hypotheses, designing key experiments and controls, and ensuring rigorous validation. Equally important, though sometimes less appreciated, are transversal components that ensure research extends its impact beyond the lab, such as open-science practices, ethics, dissemination, communication, outreach or data management. These do not only ensure scientific integrity and research quality but also make findings more accessible for diverse audiences from fellow scientists to policymakers and the public.

Here, we propose adding another transversal component: Design. When incorporated from the beginning of a project, Design serves not just as an esthetic element but as a research discipline, practice and a structured set of methods. It helps researchers to overcome major communication barriers—both in engaging with society and in fostering collaboration between disciplines that might otherwise remain isolated.

This additional “ingredient” in collaborative research is especially critical when addressing complex scientific topics. Design-driven approaches to outreach and communication can help to create innovative ways to distill these complexities into intuitive visualizations, interactive models, and narrative frameworks that make science accessible without oversimplifying. This enhances interdisciplinary collaboration and opens avenues for exploring new research directions or refining existing methodologies.

Design, in this context, is more than just a “service”. It is a structured, iterative methodology that enables researchers to visualize the invisible, clarify the abstract and communicate the complex. For scientists, it provides tools to present findings in ways that resonate with peers and stakeholders. For the public, it turns advanced molecular mechanisms into engaging stories, fostering their understanding and appreciation for the impact of scientific research. In this way, Design becomes a vital catalyst, amplifying the reach and resonance of science.

Design […] is a structured, iterative methodology that enables researchers to visualize the invisible, clarify the abstract and communicate the complex.

## ChromDesign

The Innovative Training Network Chromatin Architecture and Design, ChromDesign project exemplifies the transformative potential of embedding Design into molecular biology research. Funded under the Marie Skłodowska-Curie Actions (MSCA), ChromDesign brought together 17 institutions across 9 countries. For 4 years, the network trained 14 PhD fellows, with 13 focusing on chromatin architecture and epigenetics, while one fellow pursued a dedicated PhD project on leveraging Design to communicate these findings effectively between disciplines and society.

The ChromDesign molecular biologists made significant advances in the field of chromatin architecture, uncovering key insights into how chromatin dynamics influence gene regulation and the role of epigenetic modifications in driving cellular differentiation and disease processes.

The designer Carla Molins-Pitarch—currently a lecturer and researcher at the Image Processing and Multimedia Technology Center in DICODE—Digital Culture and Creative Technologies Research Group and former PhD fellow at Elisava Design School and Engineering—built upon these discoveries while conducting original research. Her work demonstrated that integrating Design throughout the project can expand its scope, foster novel forms of outreach, and strengthen the connections between scientific research and society, ultimately boosting the overall impact.

## Lost in translation: communicating complex research in molecular biology

In modern research, breakthroughs often emerge when researchers collaborate across disciplines (D’Este and Robinson-García, 2023; Ren et al, 2023). Yet, as scientific research has grown increasingly specialized (Fortunato et al, 2018), communicating complex concepts to others, even within the scientific community, has become a significant challenge, and molecular biology is no exception. Fields such as chromatin biology and epigenetics exemplify this issue, as they operate across multiple levels of biological organization from single genes to large-scale genome structures (Jerkovic and Cavalli, 2021). These processes are complex, dynamic, and interdependent, making them difficult to convey to specialists outside the field, let alone to policymakers, educators, or the broader public.

… as scientific research has grown increasingly specialized […] communicating intricate concepts to others, even within the scientific community, has become a significant challenge…

**Figure 1:**
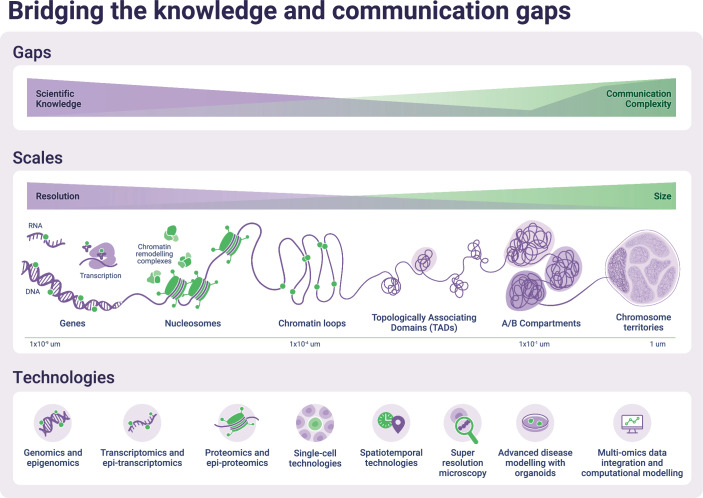
Bridging the knowledge and communication gaps through scales and technologies. Designed by Joana C. Carvalho.

Chromatin biologists’ efforts to communicate effectively are challenging owing to the vast and complex data generated through genomics, transcriptomics and proteomics technologies applied to different biological contexts and resolutions, such as patient-derived organoids studied at single-cell resolution. These methods produce massive datasets that require sophisticated bioinformatics tools for interpretation, and even experts may struggle to distill these into comprehensible narratives for other researchers. The multidimensional nature of the data, with layers of information on gene expression, protein interactions, and metabolic pathways, adds to the difficulty, making it harder to translate into the simple, relatable terms necessary to communicate to broader audiences (Marx, 2013). Moreover, chromatin operates on multiple spatial and temporal scales (Kouzarides, 2007), which adds to the difficulty of making this research comprehensible (Fig. 1).

At its most fundamental level, chromatin biology studies how DNA is packaged inside the nucleus. The DNA molecule is tightly wound around proteins called histones, forming so-called nucleosomes. This packaging determines which parts of the DNA are accessible to RNA polymerase for gene expression, influencing everything from cellular function to organism development. Histones themselves can be modified through methylation or acetylation, which act like switches that turn genes on or off. While the idea of nucleosomes and histone modifications may sound straightforward in isolation, these processes are just one part of a larger, interconnected system. As we zoom out, chromatin forms complex loops that bring distant parts of the genome into contact, enabling long-range gene regulation. These loops cluster into regions called Topologically Associating Domains (TADs), which ensure that genes and their regulatory elements interact with each other efficiently. At an even larger scale, the genome is organized into active and inactive compartments, and chromosomes occupy distinct territories within the nucleus. Together, these levels of organization create a hierarchical system that controls gene expression in time and space (Fig. 1).

**Figure 2:**
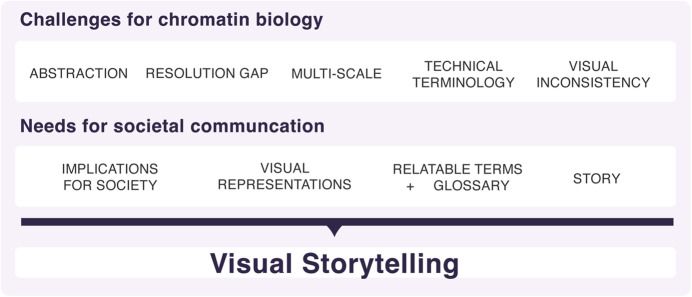
Molecular and chromatin biology issues about communication may be addressed using visual storytelling. Designed by Carla Molins-Pitarch.

This complexity creates a challenge for communication not only between scientists and the public but also among researchers from different disciplines (Fig. 2). Basic research focuses on fundamental knowledge, but often relies on abstract concepts and limited resolution, which are difficult to explain. In applied research, the emphasis on problem-solving is accompanied by specialized terminology and a lack of universal visual tools, making it harder to communicate findings to a broader audience. Together, these factors contribute to a persistent knowledge gap that prevents important discoveries from being fully appreciated and translated into potential benefits for society, patients, or public health.

In recent years, molecular biology has made significant strides in visualizing discoveries (Myers, 2015; Goodstadt and Marti-Renom, 2019). Advances in computational techniques along with new tools and approaches to visualize and analyze large amounts of data inspired the field of Biological Visualization—now a research field in its own right—to develop solutions to display the complexity of scale, abstraction of invisible processes, and large datasets (O’Donoghue et al, 2010). Visual representations act as a universal language that not only helps scientists to analyze data and recognize underlying patterns but also enables better access to knowledge by making science more approachable and compelling for diverse audiences. Yet, these tools are still limited, and novel approaches, toolkits, and insights are needed (Goodstadt and Marti-Renom, 2017). Herein are the opportunities for true transdisciplinary research that combines Design and molecular biology as a means of analyzing biological complexity from a human-centered perspective and developing new tools for efficient visual storytelling (Fig. 2).

## Design as the official translator

Design has historically contributed to the visual representation of scientific discoveries, often without explicit recognition. In “Milestones in the History of Thematic Cartography, Statistical Graphics, and Data Visualization”, Michael Friendly highlights, through a historical retrospective, the importance of the intersection of various disciplines in transforming knowledge into a visual language that generates even newer insights.

Though not considered designers in the modern sense, illustrators, cartographers, typographers, and even scientists have been essential in visually conveying knowledge. From the detailed botanical illustrations of the Renaissance to Rosalind Franklin’s interpretation of Photo 51, science has relied on visual language to convey its ideas. These contributors went beyond simple depiction, to clarify information, simplify complexity, and enhance understanding.

From the detailed botanical illustrations of the Renaissance to Rosalind Franklin’s interpretation of Photo 51, science has relied on visual language to convey its ideas.

Design encompasses an interdisciplinary research field that can bring together diverse disciplines such as art, technology and human psychology, among others, and offers tools to develop products and bridge communication gaps. By translating complex molecular concepts into accessible visual narratives, interactive experiences, and user-centric frameworks, Design can help to foster broader engagement and understanding among diverse audiences, including scientists, policymakers, and the public.

Design methodologies prioritize clarity, creativity, and user experience (Rösch et al, 2023). For example, Design-driven visualizations can illustrate how chromatin loops bring distant genomic elements together, making these abstract processes easier to grasp for non-specialist audiences (Drogaris et al, 2024). Creative technologies and storytelling can bring the complexities of chromatin biology to life through immersive exhibitions or interactive mediums. These mediums, for instance, can show how changes in the building blocks of life, at scales ranging from DNA modifications to chromatin loops and chromosome territories, can influence health and disease. By making invisible molecular structures visible, they connect audiences to complex biological processes that might otherwise seem abstract, fostering a deeper connection between science and society (Molins-Pitarch, 2023).

## What is design?

But what is Design exactly? According to the International Council of Design, it “is a discipline of study and practice focused on the interaction between a person—a “user”—and the man-made environment, taking into account esthetic, functional, contextual, cultural, and societal considerations. As a formalized discipline, Design is a modern construct.” Everyday interactions with physical objects, architectural spaces, and digital platforms shape most people’s understanding of Design. We engage with our surroundings and with others through these designed elements. Everything from clothing and gadgets to transportation systems, user interfaces, landscapes, cities, and even the chair you’re sitting on has been conceived by a designer.

A great example is the smartphone, where multiple Design disciplines converge. Industrial Design creates the phone’s form, UI/UX Design ensures an intuitive user experience, and Graphic Design focuses on visual elements such as icons and layouts. Interaction Design improves how users physically engage with the device, and Service Design extends to customer support and app stores. These disciplines come together to create a functional product that influences how we interact with technology, much like biological systems that integrate various functions. The astounding success of the iPhone when it first became available was in good part because Apple has deeply integrated Design at the forefront of its development and engineering processes.

**Figure 3:**
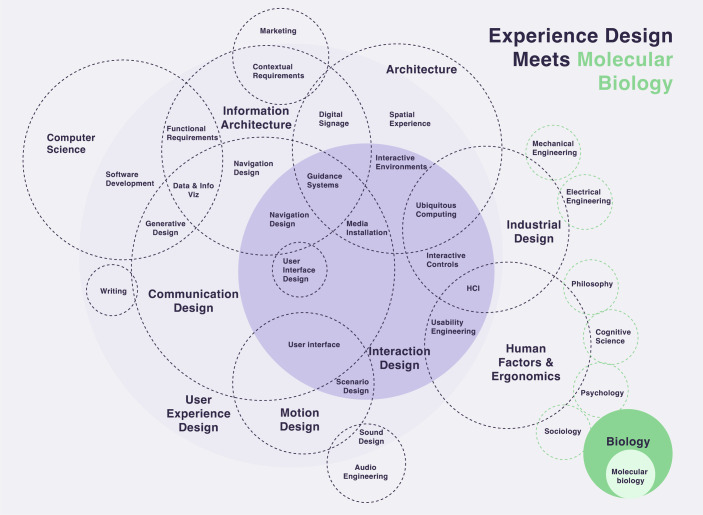
Experience design disciplinary relationships toward molecular biology. Custom graphic based on Hooper and Dix, 2012. Designed by Carla Molins-Pitarch.

In that way, Design is not just merely about esthetics but a discipline connector as it can intersect with multiple and diverse fields such as psychology, computer science, and even molecular biology (Fig. 3). For instance, Design uses psychology to make interfaces intuitive and to reduce cognitive load, while computer science ensures smooth hardware-software integration. Even biological principles, like biomimicry, inspire innovative, ergonomic designs (Myers, 2015). Overall, Design is a powerful, interdisciplinary tool that shapes how we think, behave, and interact with the world.

Design is a powerful, interdisciplinary tool that shapes how we think, behave, and interact with the world.

Design research is, therefore, essential as it requires a deep understanding of users’ needs and behaviors and addresses the context in which they interact with products and systems. Design research ensures that solutions are user-centered and effectively address real-world problems by incorporating methods from other disciplines. This research-driven approach helps designers make informed decisions, leading to products, services and processes that are more intuitive and efficient (Norman, 2013).

Design research ensures that solutions are user-centered and effectively address real-world problems by incorporating methods from other disciplines.

In addition, Design research fosters innovation by uncovering unmet needs and emerging trends to inspire the development of novel solutions. It also promotes sustainability and ethical practices by considering the long-term social and environmental impacts of design choices. Overall, Design research is a critical component for creating products and systems that are not only visually appealing but also benefit users and society (Buchanan, 2001).

Why is Design research so relevant in the case of chromatin biology? Design practices and processes transform abstract biological concepts into tangible experiences, while science communication makes these ideas accessible to a broader audience. This synergy enhances scientific understanding and public engagement and transforms abstract scientific ideas into comprehensible, relatable outputs, enabling molecular biology to transcend traditional disciplinary silos and communicate its discoveries with clarity and impact. Such a partnership not only amplifies the societal relevance of biological research but also redefines how we share science in an interconnected world. Design is a young, intersectional field but with the potential to resolve a communication conundrum.

Beyond its role in science communication, Design also influences the very architecture of research. By integrating Design Thinking into experimental planning and data analysis, researchers can structure experiments in innovative ways that reveal relevant insights while fostering creative problem-solving. This converges with the concepts of Day and Night Science, where rigorous planning meets innovative experimentation, ultimately positioning Design as a dynamic connector across all stages of research that can help spark new disruptive ideas (Yanai and Lercher, 2019, 2023).

## Introducing design to molecular biology through transdisciplinarity

Traditionally, experimental research aims at testing a theory to develop a clearer understanding of how the world operates. Similarly, designers prioritize understanding the context and creating effective systems to drive meaningful change. The objective of design-based research is to refine both theory and practice through the research process (Armstrong et al, 2020), making it highly complementary; for instance, both molecular biology and Design are driven by curiosity, exploration, and the desire to understand and improve the world.

Design-based research is both collaborative and iterative, producing two types of outcomes: tangible and practical or intangible and theoretical. Tangible outcomes are products and services for users and customers. Intangible outcomes generate new knowledge that informs future research and practice. The research process is flexible and can evolve iteratively to suit specific contexts, but it always involves stages of analysis and exploration, design and construction, as well as evaluation and reflection (Barab and Squire, 2004; Collins et al, 2004). Design research’s iterative problem-solving and prototyping complements the experimental approaches in research to find an innovative approach to communicating science as a tangible outcome and generating new processes and systems as intangible ones. Table 1 lists some of the universal Design methods that support this particular Design research process for communicating molecular biology.

**Table 1:** Universal methods of design (Martin and Hanington, 2012) selection to research complex problems in communicating molecular biology.

**Creative toolkits**	Creative toolkits are curated sets of physical components designed to facilitate participatory modeling, visualization, or imaginative exploration, enabling users to inspire and inform design and business teams effectively.	**Case studies**	A case study is a research approach that involves an in-depth examination of specific events or instances within their context, drawing on multiple sources of evidence to gain comprehensive insights.
**Design workshops**	Design workshops are structured participatory sessions that bring together multiple participants and design team members, utilizing collaborative co-design methods to foster creativity and joint problem-solving.	**Participant observatio**n	Participant observation is an immersive ethnographic method that involves engaging as a member within an activity, context, culture, or subculture to gain a deeper understanding of situations and behaviors through firsthand experience.
**Mind mapping**	Mind mapping is a visual thinking technique that facilitates idea generation and concept development, especially when relationships between various pieces of related information are not immediately clear.	**Prototyping**	Prototyping involves creating tangible artifacts at different levels of detail to develop and test ideas, both within design teams and in collaboration with clients and users.

During the last few years, a group of academics and practitioners have tried to set the foundations for Design research. Ehn and Ullmark define a “designerly way of managing complexity” that can complement other forms of knowledge development. They argue that a process-oriented perspective often falls short of meeting the needs of designers and design theorists, who typically focus on systems or final products. Moreover, interactive design must integrate insights from other academic disciplines, fostering a cross- and transdisciplinary approach. For the outcomes to be valuable to other Design researchers, it is essential to strike a balance between adapting theory from other fields and its practical applications (Vaughan, 2020). Undoubtedly, this “designerly way of managing complexity” is one of the keys to generating the expected impact, demonstrating the potential for design to create accessible narratives and artifacts that bring complex biological processes to life for varied audiences.

All in all, it seems that synergy among disciplines is key to finding new potential solutions to communicate complexity. So, how is it that these frameworks are not more widely used already? It is important to note that efficient collaboration between disciplines involves solidarity, trust, and support among all its members. There is a need for advocacy for the incoming discipline to be recognized and treated equally to achieve a maximal impact (Kelley and Kelley, 2013).

There is a need for advocacy for the incoming discipline to be recognized and treated equally to achieve a maximal impact.

There are many ways to collaborate across disciplines, but a truly transdisciplinary approach creates an environment where boundaries blur, new research questions arise, and fresh breakthroughs become possible. Building such a partnership often involves a range of contributors—including scientists, designers, policymakers, and the public—each bringing different perspectives and expertise. To show how this can work in practice, the ChromDesign project fully integrated Design research into a molecular biology framework as a compelling example of transdisciplinary collaboration.

## Re-designing the boundaries of research and outreach

**Figure 4:**
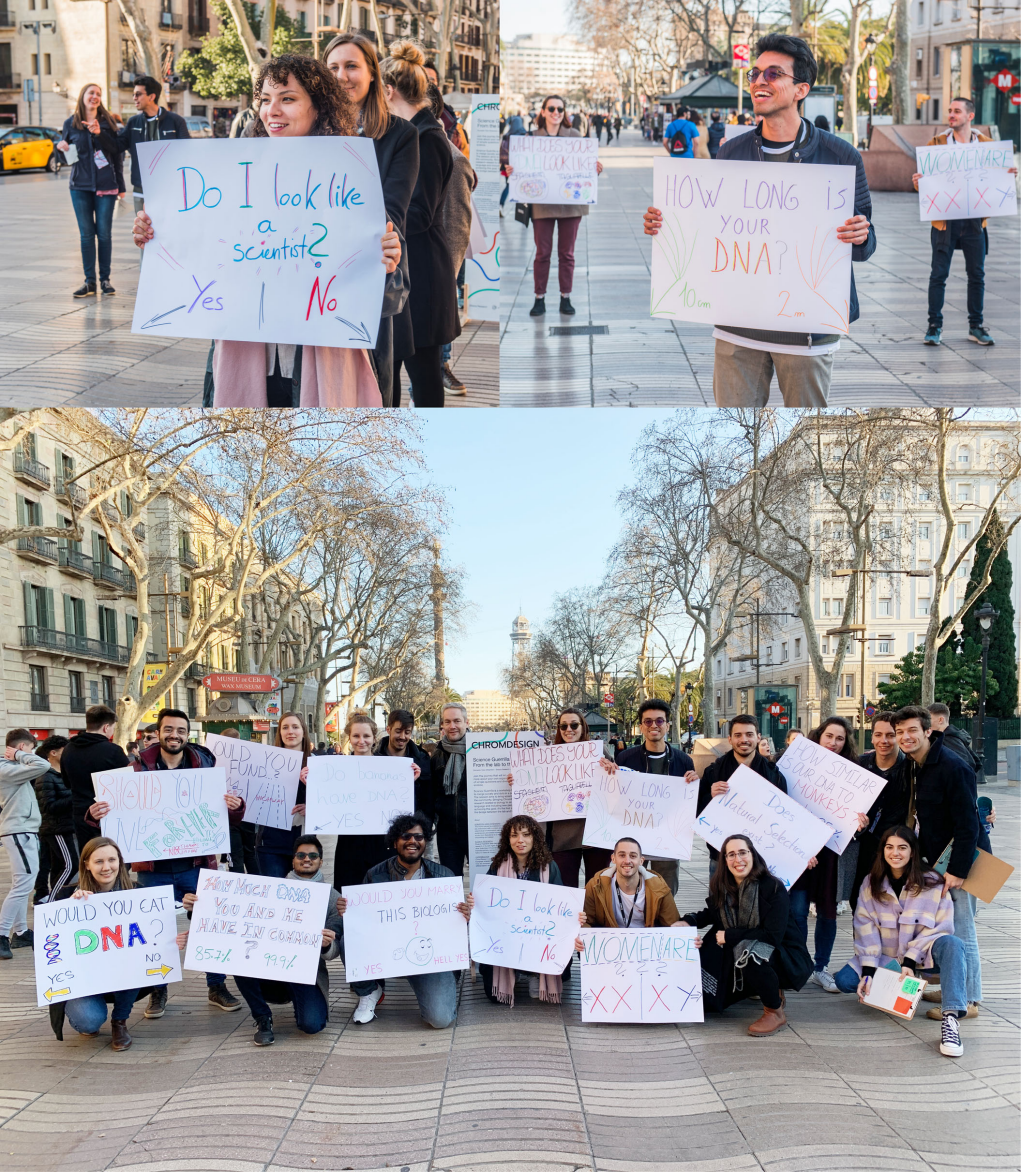
Science Guerrilla in action at Las Ramblas, Barcelona. The Science Guerrilla is an impromptu public space action where each participant created a hand-drawn poster including a DNA-related question with the goal to attract random pedestrians in a busy public space and spark spontaneous conversations relating to their research topics. Pictures by Ardila Photo Studio and Jonas Krebs.

In any new project, you need to evaluate existing methods and see if they fit your current needs. The ChromDesign project required ad hoc processes to ensure all its goals: specialized design training for molecular biologists, the creation of a collaborative ‘toolkit’, and public engagement activities. One cornerstone for bridging molecular biology and Design within the network was the Transdisciplinary Communication Training School, conducted during the first 6 months of the fellows’ PhD projects. This intensive program introduced participants to Design as a research method, as a communication tool and as a bridge between disciplines. Workshops included Design Thinking for Scientists, Visualizing the Invisible, and Storytelling Techniques, providing PhD fellows with innovative strategies to make complex biological concepts accessible via compelling narrative. These foundational skills catalyzed many of the network’s subsequent activities and enabled PhD fellows to develop innovative communication tools that reimagined how chromatin biology could be shared with diverse audiences. Key public engagement activities of ChromDesign include the Science Guerrilla workshop, where fellows took their research to Barcelona’s Las Ramblas in 2020, directly engaging with citizens through interactive dynamics (Fig. 4).

**Figure 5:**
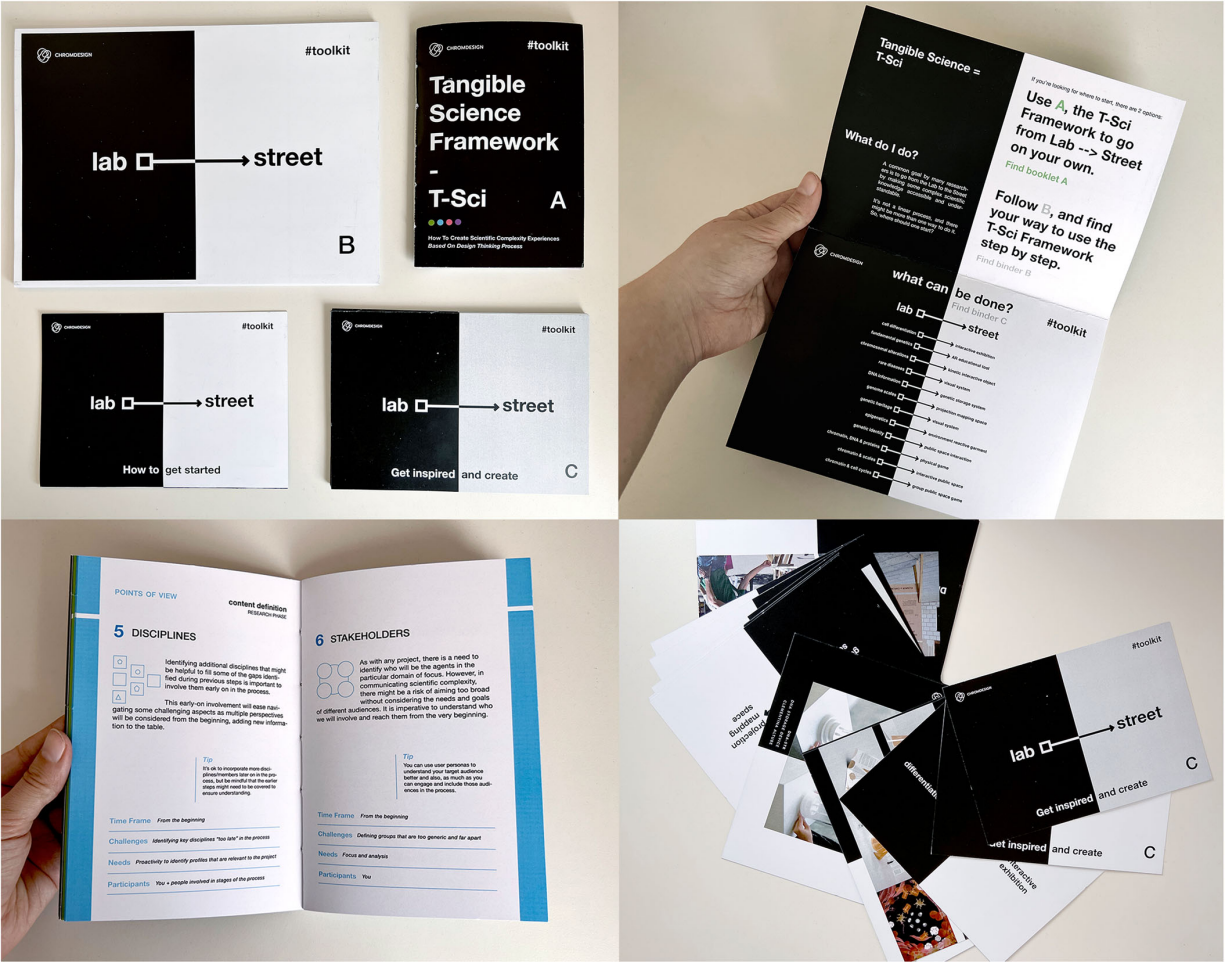
The Toolkit: Lab to Street, Tangible Science Framework. The Toolkit is divided into different parts (A–C) that help to integrate the framework into any team that wants to take scientific concepts closer to society. In the different pictures depicted: ‘How to get Started’ leaflet, the T-Sci Framework itself (A), Step by Step Start (B) and a booklet of resulting projects as a source of inspiration (C).

**Figure 6:**
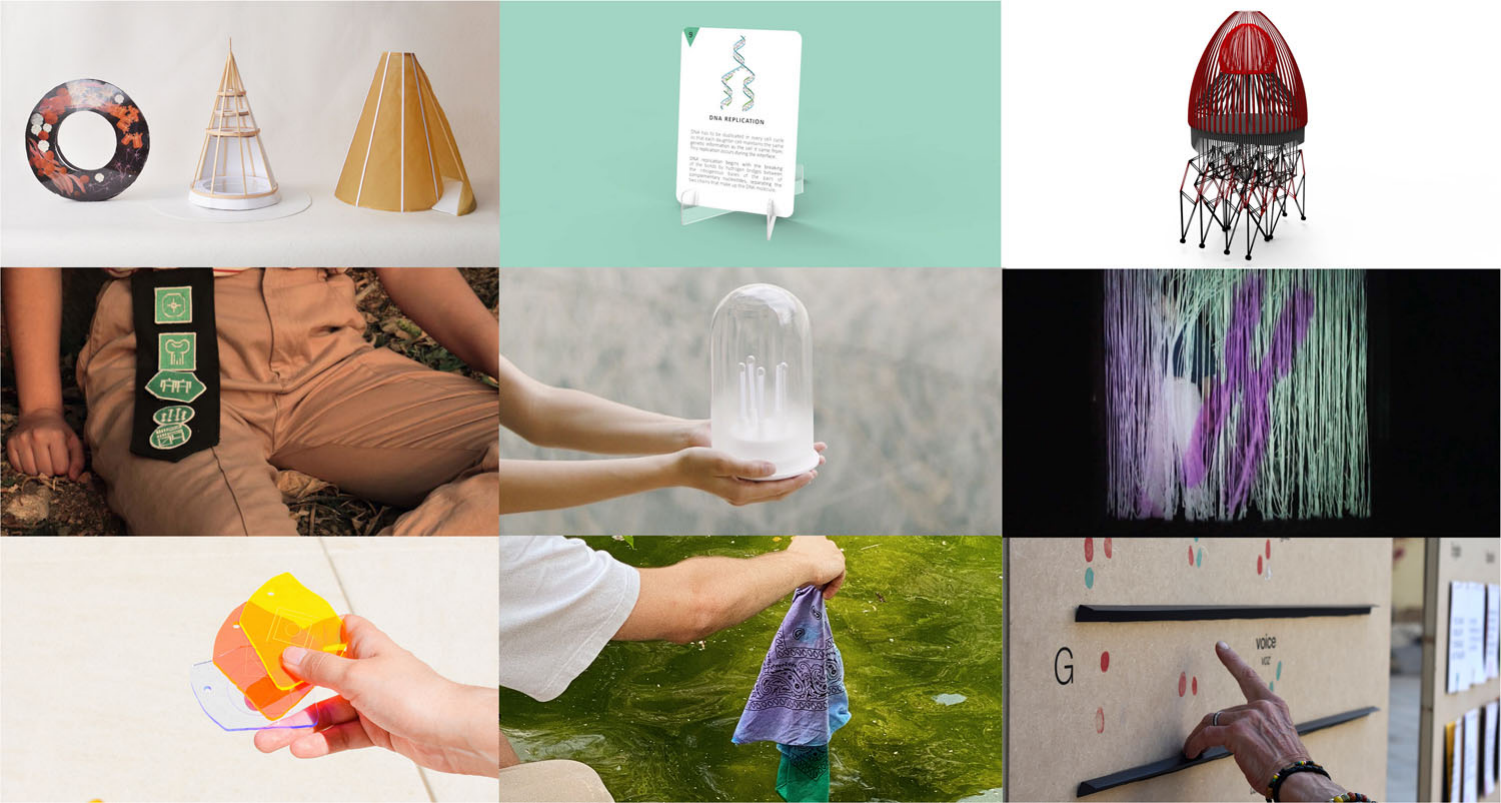
Design and Design Engineering student final degree projects. ELISAVA students: Javier Domingo, Mónica Pérez, Joan Victòria, Yolanda Justicia, Clementina Altube, Santi Bonet, Xin Ye, Toni Bové, Judit Castells.

We also identified the need for new communication tools for collaboration between all ChromDesign stakeholders. The Toolkit: Lab to Street, also known as the Tangible Science Framework (T-Sci), is a hybrid approach that combines theoretical and practical elements (Fig. 5). It provides a shared framework to promote transdisciplinary collaboration, the exchange of methodologies, lexicons, checkpoints, and progress toward a common goal—improving public communication—among researchers from diverse disciplines. In brief, the toolkit supports a Design process for researchers familiar with Design methodologies who aim to bridge the gap between science and society. It facilitates collaboration among scientists and stakeholders, incorporating diverse perspectives (Molins-Pitarch, 2021). Thanks to this new toolkit, 19 projects to communicate ChromDesign science were created, including 9 Design undergraduate final projects during the span of 3 years, to experiment on how to communicate molecular biology’s complexity (Fig. 6). Projects ranging from a cell-cycle immersive exhibition, a Genetics Augmented Reality (AR) educational tool, epigenetics reactive garments, and a rare-diseases visual system resulted from this long-term collaboration, and all results have been shared in public events and conferences through a 10-minute audiovisual piece.

**Figure 7:**
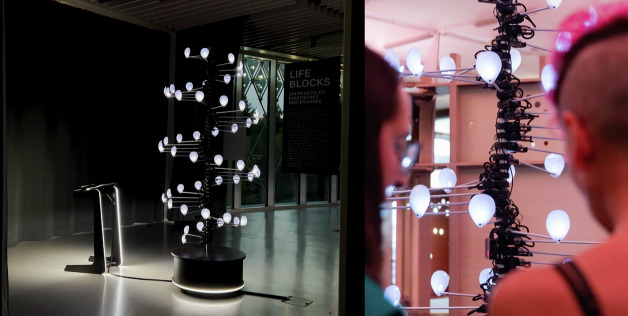
Life Blocks interactive piece in two different settings: life’s Journey Exhibition (Novartis Pavillon, Basel) and Sonar +D Weird Science area. Pictures by Carla Molins-Pitarch and Sonar +D social media.

**Figure 8:**
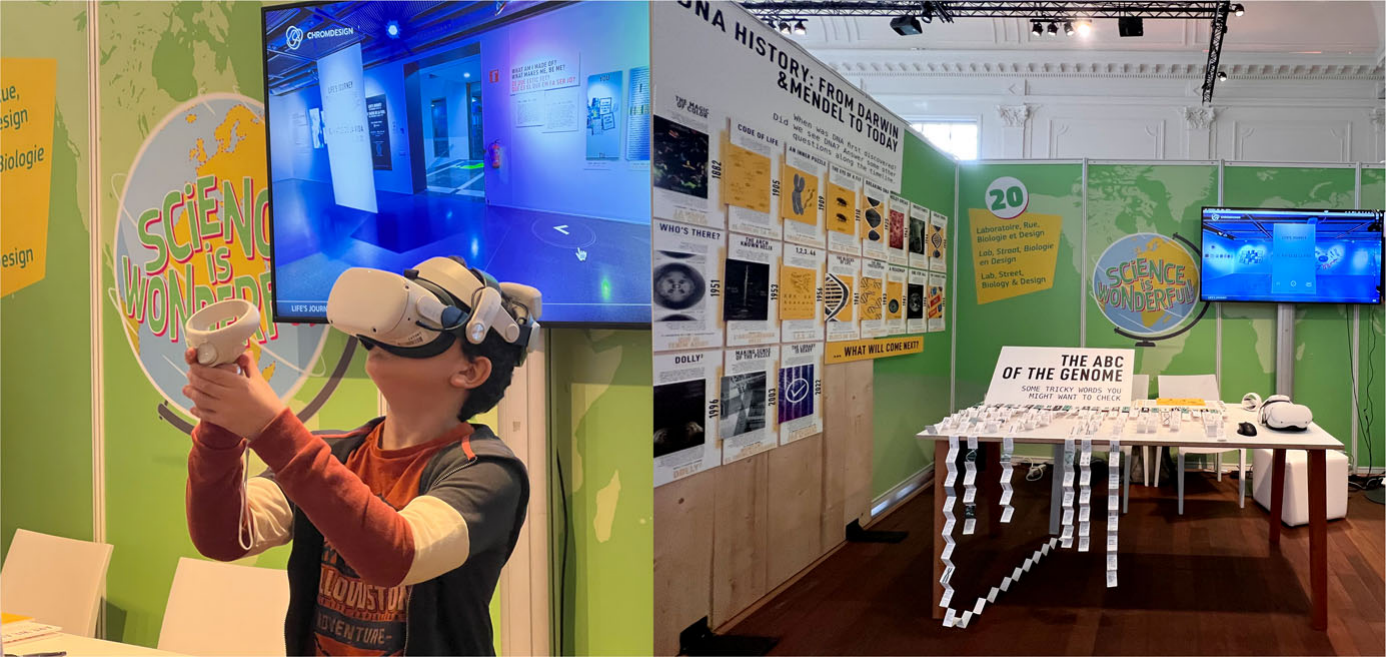
Lab, Street, Biology & Design booth at the “Science is Wonderful!” 2023 MSCA event in Brussels. Pictures by Carla Molins-Pitarch.

The ultimate tangible outcome and standout achievement was the Life’s Journey exhibition—an immersive showcase illustrating chromatin structure and its role in cellular processes through storytelling and creative technology (Fig. 7). It featured Life Blocks, an interactive modular experience to explain, learn and play with complex genomics, in particular 3D genomics. After its premiere in Barcelona at the Elisava School of Design and Engineering, and the Barcelona Biomedical Research Park (PRBB), the exhibition traveled across Europe to the Novartis Pavillon in Basel, the Science is Wonderful event in Brussels, and other research centers and R&D festivals such as Sonar +D, reaching more than 6000 researchers, designers, and non-experts (Fig. 8). The exhibition remains accessible online and in 3D virtual reality.

These efforts were supported by a robust communication strategy, featuring, for example, formal and informal videos of the PhD fellows’ research projects and a network blog that amplified ChromDesign’s outreach beyond academia. The project also explored Design-driven activities that inspired potential new research lines in the field of 3D genomics and Biological Visualization (Molins-Pitarch, 2023).

ChromDesign’s interdisciplinary approach enriched all participants, equipping PhD fellows with unique skills for communicating complex science through Design, while supervisors and project managers gained new perspectives on collaborative research. For the molecular biology community, Design methodologies introduced fresh ways to conceptualize chromatin dynamics. Conversely, designers engaged with real-world scientific challenges, demonstrating how creative practices can deepen understanding and amplify impact. This novel fusion of Design and molecular biology was clearly embodied in the network’s final in-person meeting in the form of an international conference of chromatin biology—EpIC Conference, Granada, 2022—where a scientific poster session shared space with a science-inspired art exhibition and an artistic microscopy photo contest, together with interactive workshops led by both designers and molecular biologists—blurring disciplinary boundaries while celebrating creativity and collaboration. Among the showcased projects was INSIDE-OUT: KNITTED DNA, an artist-in-residence collaboration between ChromDesign and Carolin Vogler, funded by the STARTS initiative. This installation merged science, fashion, and technology by transforming genetic information into wearable artworks, allowing visitors to engage with DNA structures through textiles and explore the visual esthetics of genetic variations across species.

For the molecular biology community, Design methodologies introduced fresh ways to conceptualize chromatin dynamics.

The broader influence of ChromDesign extends beyond its immediate participants and immediate outcomes, strengthening connections between science and society. By translating chromatin biology into accessible narratives and interactive experiences, the project sparked curiosity and engagement, demonstrating the potential of transdisciplinary partnerships to bridge gaps, inspire innovation and redefine the boundaries of research and outreach.

## Lessons learned: a lifelong alliance for science communication

ChromDesign showed how combining scientists, designers, and communication experts can address challenges in communicating cutting-edge research. By integrating biology with Design, it demonstrated new ways to study, present and understand complex concepts, offering lessons for future initiatives.

One clear lesson is that well-structured, yet flexible teamwork is crucial. Shared goals, openness among participants, and defined roles maintained scientific quality while encouraging creativity. Project managers and communication specialists played key roles in keeping the work organized and engaging. This environment allowed scientists to use Design methods to clarify their findings, while designers gained deeper insights into biological processes—proving Design is more than just a “service” but rather a meaningful part of research.

As Tina Karagyozova—currently a postdoctoral researcher at the University of Edinburgh and a former ChromDesign PhD fellow at the Institut Curie’s Chromatin Dynamics group—commented, “My main takeaway has been that this collaboration highlighted the similarities between the Design and scientific approach like iterative testing/experimentation, and that this focus on continuous improvement and learning from experience is crucial in learning how to better communicate science to the public.” Her perspective encapsulates the essence of ChromDesign, where the fusion of iterative practices in both design and science fosters an environment of continuous learning and improvement.

ChromDesign also pointed to a broader shift in how science can be communicated. In areas such as chromatin biology, where processes can be difficult to visualize, Design approaches—such as immersive storytelling or interactive graphics—make research more accessible and appealing to both experts and the wider public. Viewing Design as an equal partner opens the door to new ideas, breaks communication barriers, and helps discoveries reach their full potential.

We encourage other research networks to draw on ChromDesign’s experience by forging long-term collaborations that integrate experts from diverse fields, including the Arts and Humanities. The success of ChromDesign illustrates that Design can act as a powerful connector—enhancing the way we address complexity and communicate challenging ideas. In specialized fields, where advanced research and complex technologies can be challenging for those outside the discipline to navigate, this approach can foster unexpected research lines and generate richer interdisciplinary dialogs. Design can bridge the gap between laboratories and everyday life, shaping how we experience, understand, and value scientific research. By embracing Design as a bridge between disciplines, research initiatives can develop novel collaborative frameworks that not only advance scientific inquiry but also bring science closer to society. Ultimately, the true power of science lies not only in making groundbreaking discoveries but also in our ability to connect with, inspire, and transform society.

The success of ChromDesign illustrates that Design can act as a powerful connector—enhancing the way we address complexity and communicate challenging ideas.

Ultimately, the true power of science lies not only in making groundbreaking discoveries but also in our ability to connect with, inspire and transform society.

## Supplementary information

Peer Review File
