# Supplementary material for: Molecular biology and design research: A new paradigm and transdisciplinary approach to communicating science
Source: EMBO Rep. 2025 Mar 31;26(8):1913–22. doi: 10.1038/s44319-025-00434-4 (PMC12019188; doi:10.1038/s44319-025-00434-4)
Supplement: Supplementary file 1 — Peer Review File [file 44319_2025_434_MOESM1_ESM.pdf]

EMBO reports

Molecular biology and Design research

Carla Molins-Pitarch, Jonas Krebs, and David Brena

Corresponding author(s): David Brena (david.brena@crg.eu) , Jonas Krebs (jonas.krebs@crg.eu), Carla Molins-Pitarch (carla.molins@citm.upc.edu)

Review Timeline:

Submission Date:	11th Mar 25
Accepted:	14th Mar 25

Editor: Holger Breithaupt

Transaction Report: This article was editorially reviewed.
